# Pursuing nurses’ work effectiveness and better hand-hygiene compliance in a intensive care unit (ICU) ward: application of lean methodologies

**DOI:** 10.1186/2047-2994-4-S1-P281

**Published:** 2015-06-16

**Authors:** J Gregório, I Santos, F Pinheiro, P Póvoa, L Lapão

**Affiliations:** 1 WHO Collaborating Center on Health Workforce Policy and Planning, Lisboa, Portugal; 2 Instituto de Higiene e Medicina Tropical, Lisboa, Portugal; 3 Intensive Care Unit Ward, S. Francisco Xavier Hospital, Lisboa, Portugal; 4 Nova Medical School, Lisboa, Portugal

## Introduction

Healthcare acquired infections (HAI) are an important cause of morbidity and mortality. Hand hygiene (HH) is one of the most effective measures for preventing HAI. However, stirring healthcare workers to comply with HH remains a challenge. Two common barriers often found are forgetfulness and the lack of time.

## Objectives

We hypothesize that the perception of lack of time and forgetfulness stems from the pressure of too much tasks in a short period of time. Therefore, it is rational to search for efficiency and minimize waste. This study aims at exploring the use of LEAN methodologies to improve nurses’ work processes in an ICU ward.

## Methods

A design science research approach was used. A questionnaire was applied to the nurses followed by an observational study of an 8-hour work shift to identify nurses’ activities, processes of care, and to describe HH compliance. Next, a value stream map (VSM) was designed and the analysis performed with the nurses, which helped to identify points of possible improvement in the process of care. After this, weekly LEAN workshops were held with the objective of improving workflow efficiency.

## Results

From the initial observational study, we found that a nurse may take on average 16% of their work time using the information system, and the overall rate of HH was 63%. The full compliance to HH would amount to 13% of a nurse’s workload. Three processes were identified as the most relevant HH compliance drivers: the reorganization of essential supplies, the optimal provision of medicines and monitoring equipment automatic data collection. The participant nurses defined as objectives to reduce the supplies’ delivering time and establish a stock management system. To achieve it the reorganization of the storage with the ABC method, with the nurses deciding the layout of the storage space, was essential.

## Conclusion

The use of LEAN methods permitted to detect and address improvement opportunities with significant benefits. Moreover, nurses’ participation in the process enriched the experience, helping to design a customized intervention and heightened their sense of teamwork.

## Disclosure of interest

None declared.

